# Photo and Plasma Activation of Dental Implant Titanium Surfaces. A Systematic Review with Meta-Analysis of Pre-Clinical Studies

**DOI:** 10.3390/jcm9092817

**Published:** 2020-08-31

**Authors:** Paolo Pesce, Maria Menini, Gregorio Santori, Emanuele De Giovanni, Francesco Bagnasco, Luigi Canullo

**Affiliations:** 1 Department of Surgical Sciences and Integrated Diagnostics, University of Genoa, 16132 Genoa, Italy; paolo.pesce@unige.it (P.P.); maria.menini@unige.it (M.M.); gregorio.santori@unige.it (G.S.); lele9-90@hotmail.it (E.D.G.); fcbagna5@hotmail.it (F.B.); 2 Private Practice, Via Nizza, 46, 00198 Rome, Italy

**Keywords:** dental implants, UV photofunctionalization, non-thermal plasma functionalization, osseointegration

## Abstract

Background: Ultraviolet (UV) and non-thermal plasma functionalization are surface treatment modalities that seem able to improve osseointegration. The aim of this systematic review and meta-analysis is to assess the effect of the two methods and possible differences. Materials and Methods: The systematic research of pre-clinical animal studies was conducted up to May 2020 in the databases PubMed/Medline, Scopus and the Cochrane Lybrary. A meta-analysis was performed by using the DerSimonian–Laird estimator in random-effects models. Results: Through the digital search, 518 articles were identified; after duplicate removal and screening process 10 papers were included. Four studies evaluating UV treatment in rabbits were included in the meta-analysis. The qualitative evaluation of the included studies showed that both UV photofunctionalization and non-thermal plasma argon functionalization of titanium implant surfaces might be effective in vivo to improve the osseointegration. The meta-analysis on four studies evaluating UV treatment in rabbits showed that bone to implant contact values (expressed as standardized mean differences and raw mean differences) were significantly increased in the bio-activated groups when follow-up times were relatively homogeneous, although a high heterogeneity (*I*^2^ > 75%) was found in all models. Conclusions: The present systematic review and meta-analysis on pre-clinical studies demonstrated that chair-side treatment of implants with UV or non-thermal plasma appear to be effective for improving osseointegration. This systematic review supports further clinical trials on this topic.

## 1. Introduction

Prosthetic rehabilitation with dental implants represents a successful therapy for the replacement of severely compromised or missing teeth, with long-term success rates above 95% [1]. The success of dental implants largely depends on a safe integration into the maxillary bone, or rather achieving stable osseointegration over time [2]. Osseointegration is considered a demarcation response to a foreign body of Ti when the Ti implant is immobile in bone [3]. This demarcation is immune-driven and is classified as a type IV hypersensitivity [4]. Based on the original definition, the modification of a Ti implant surface implies that the surface would be more biocompatible, thereby increasing the bioaffinity of the hard tissue and accelerating the bone response to the surface. In order to improve the biological response to Ti implants, various techniques have been suggested in order to modify Ti surface roughness, chemistry, topography, and electrical charge, focusing on the biological performance of Ti surfaces [5,6,7] Higher bone–implant contact (BIC) values, better bone apposition and peri-implant bone maintenance over time were demonstrated on implants with rougher surfaces compared to machined surfaces, including stimulation of cell migration and proliferation [6,7,8]. BIC values in modern implants normally vary between 65 and 73% but do not reach the ideal 100% [9]. Moreover, titanium surface modifications showed, in short-term evaluations, an enhanced connective fiber attachment and a similar inflammatory response [10,11].

In recent years, various methods (ultraviolet (UV), non-thermal plasma functionalization, blasting, etching, and anodization) were proposed to improve the hydrophilicity of the titanium surface, its functionality and in order to improve its chemistry as well as reduce surface contamination [5,12]. Among them, (UV) and non-thermal plasma functionalization are surface treatment modalities that are able to improve all the biological aspects mentioned above and can be applied chair-side [11,13]. They allow maintaining the micro- and nano-topographical features of titanium surfaces, enhancing the biological potential before implant surgery, without damaging the surface topography. Various in vitro studies about UV and non-thermal plasma functionalization showed that this method is able to increases hydrophilicity, turning the electrostatic charge to positive, and removing hydrocarbons from the surface. Positive effects are induced also on alkaline phosphatase activity, calcium deposition, spreading of human stem cells, protein absorption capacity, osteoblast migration, attachment, spread, and proliferation [13,14,15,16,17].

The aim of this systematic review was to assess in pre-clinical animal studies the effect of UV photofunctionalization or non-thermal plasma functionalization on the osseointegration of dental titanium implants.

## 2. Material and Methods

This systematic review fully adhered to the guidelines of the preferred reporting of systematic reviews and meta-analyses (PRISMA) statement [18] and the protocol was registered on Prospero (CRD42020185209). The proposed focused question was: “What is the effect of UV photofunctionalization and non-thermal plasma of argon activation of titanium dental implants on osseointegration in animals?” The focused question was established according to the PICO strategy:

- Population: Healthy animals with at least one titanium dental implant.
- Intervention: Any surface activation with UV or non-thermal plasma.
- Comparison: Any type of “non-activated” titanium dental implant.
- Outcomes: Bone-to-implant contact (BIC), implant stability quotient (ISQ) or removal torque.

### 2.1. Search Strategy

An electronic search in PubMed^®^/MEDLINE, Scopus, and the Cochrane Central Register of Controlled Clinical Trials (CENTRAL) databases was performed starting April 2020. No publication year nor language limit was applied, so that the search could include all the available papers until 21 May 2020. The search was complemented by manual searches of the reference lists of all full-text articles selected. The following search terms were used: “photofunctionalization”, “photofunctionalized”, “ultraviolet(s)”, “uv”, “plasma(s)”, “argon”, and “dental implants”. More details on research queries and Boolean operators are available in the electronic supplementary material (Table S1).

### 2.2. Eligibility Criteria

All articles on animals that presented a test group and a control group were considered eligible. Studies were required to have recruited a minimum of five healthy animals, have at least one titanium dental implant and have a minimum follow-up of two weeks. The studies were required to have compared implants treated with UV or non-thermal argon plasma (test group) and untreated implants. In addition, the studies to meet the inclusion criteria were required to have assessed the outcomes of interest (BIC value, removal torque and ISQ). Studies that did not met all the above-mentioned inclusion criteria were excluded. Review studies, retrospective studies, report studies based on questionnaires and interviews, studies without a clinical evaluation, case reports and redundant articles, studies on mini-implants and/or for orthodontic anchoring, and genomic and/or epigenomic analysis studies were also excluded.

### 2.3. Selection of Studies

Two reviewers (P.P. and E.D.G.) independently read titles and abstracts of the entries yielded from the initial electronic database search. After this initial assessment, both reviewers read separately the full-text versions of the studies that could be potentially included in this systematic review. The final selection of articles was made on the basis of the eligibility criteria described above. Any disagreement in the final selection was resolved by open discussion between both reviewers. In the case that no agreement could be reached, one of the co-author (L.C.) acted as arbiter.

### 2.4. Data Extraction

Data from the studies included in the final selection were extracted by one of the authors (E.D.G) using the Microsoft Excel spreadsheet software (Excel 16.4, Microsoft CO, Redmond, WA, USA). The accuracy of data was verified independently by another coauthor (P.P.). The following data were extracted were study design, title, author, publication year, follow-up period, number of patients (animals) and implants, implant design and surface characteristics, and the outcomes of interest (BIC, ISQ and removal torque). If data were missing, the authors of the original articles were contacted and asked to provide further details.

### 2.5. Quality Assessment

The overall quality of evidence at the outcome level in animal studies was independently assessed by two authors (E.D.G and P.P.), according to SYRCLE’s RoB tool (Systematic Review Centre for Laboratory Animal Experimentation). Risk of bias in individual studies was assessed independently and in duplicate by the two coauthors as part of the data extraction process. This evaluation was conducted using the Cochrane-recommended approach for assessing risk of bias in animal intervention studies [19], including ten quality parameters: sequence generation, baseline characteristics, allocation concealment, random housing, blinding, random outcome assessment, incomplete outcome data, selective outcome reporting, and other sources of bias. Disagreements were discussed in order to aim for consensus. Each parameter was rated as: yes (low risk of bias); no (high risk of bias); or unclear (unclear risk of bias).

### 2.6. Statistical Analysis

The statistical heterogeneity among studies was expressed as *τ*^2^ and estimated by the Cochran’s *Q* test. The *I*^2^ was calculated to assess variability due to heterogeneity rather than chance (*I*^2^ = 25%: low; *I*^2^ > 25% and = 50%: moderate; *I*^2^ > 50% and = 75%: considerable; *I*^2^ > 75%: high heterogeneity). *H*^2^ was calculated as the ratio between total and sampling variability. Maximum likelihood (ML) and restricted maximum likelihood (RML) with Akaike information criteria (AIC) were returned for model fit statistics. The estimates of the effects were expressed as standardized mean difference (SMD) or raw mean difference (RMD). Study estimates were pooled with the random effects model and the DerSimonian–Laird estimator. In the random-effects models, the selected studies and their outcomes are assumed to be a random selection from a larger population of studies. A forest plot was created for each measured outcome to illustrate the effects in the meta-analysis of the different studies and the global estimation. Contour-enhanced funnel plots with Kendall’s Tau and Egger’s regression were used for publication bias assessment. For further evaluation of residual heterogeneity, a normal quantile–quantile (Q–Q) plot was evaluated. Statistical significance was assumed in each test with P value < 0.05. Statistical analysis was carried out by using the R software (version 3.6.3; R Foundation for Statistical Computing. Vienna, Austria) with the metafor package (version 2.1-0).

## 3. Results

### 3.1. Bibliographic Search and Study Selection

**Figure 1:**
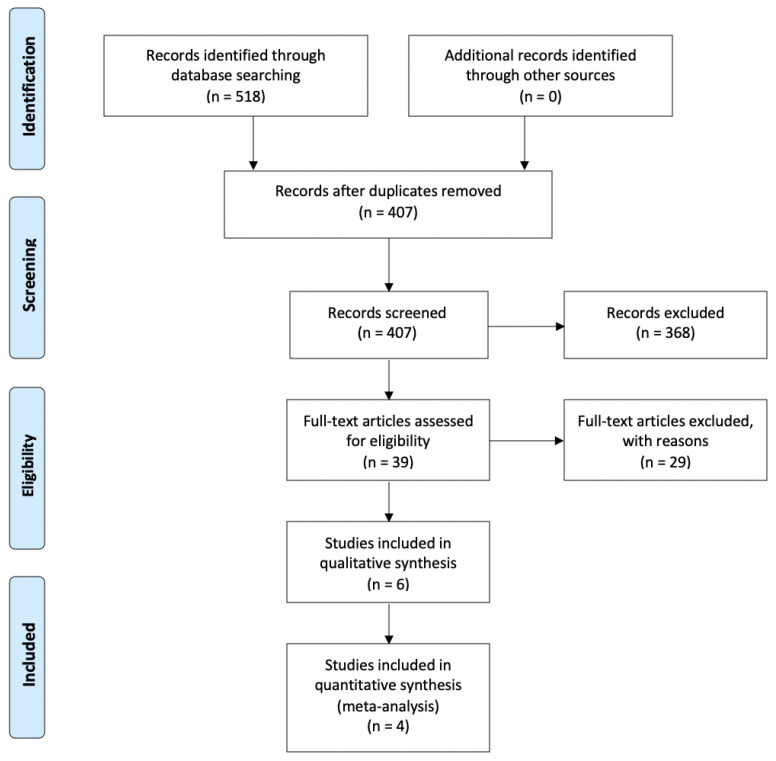
Preferred reporting of systematic reviews and meta-analyses (PRISMA) flow diagram related to bibliographic searching and study selection.

The initial database search yielded a total of 518 entries; of which, 220 were found in PubMed^®^/MEDLINE, 285 in Scopus, and 13 in Cochrane Library. A flow chart that depicts the screening process is displayed in Figure 1. After excluding all duplicates, the total number of entries was reduced to 407. A total of 368 articles were excluded after review of title and abstract. Hence, full-text examination was conducted for 39 articles. A total of 28 additional articles were excluded after full-text review and application of the eligibility criteria. The final selection consisted of 10 articles, of which 4 were included in the meta-analyses. Detailed data for the 10 included studies are listed in Table 1 and Table 2. Four authors were contacted to obtain missing information, and only one of them [20] answered.

### 3.2. Description of Included Studies

Detailed data for the 10 included studies are listed in Table 1 and Table 2. All studies included in the present review are studies performed on test case animals, with a study group and a control group. Four studies were performed on rabbits [20,26,27,29] and one on rats [28]. Five studies were performed on beagle dogs [21,22,23,24,25] The studies included in the review showed wide variations regarding length of the follow-up. The majority of them had a follow-up of a few weeks only and only two had a follow-up up to 12 weeks [25,27].

### 3.3. Excluded Studies

Out of the 39 papers for which the full-text was analyzed, 29 articles [13,30,31,32,33,34,35,36,37,38,39,40,41,42,43,44,45,46,47,48,49,50,51,52,53,54,55,56,57] were excluded from the systematic review (Table A1). The main reasons for exclusion were the following: small sample size, (articles that recruited less than five animals); outcome, (articles that did not evaluated BIC value, removal torque, or ISQ); and specimens, (articles that did not evaluated titanium dental implants).

### 3.4. Quality Assessment

**Figure 2:**
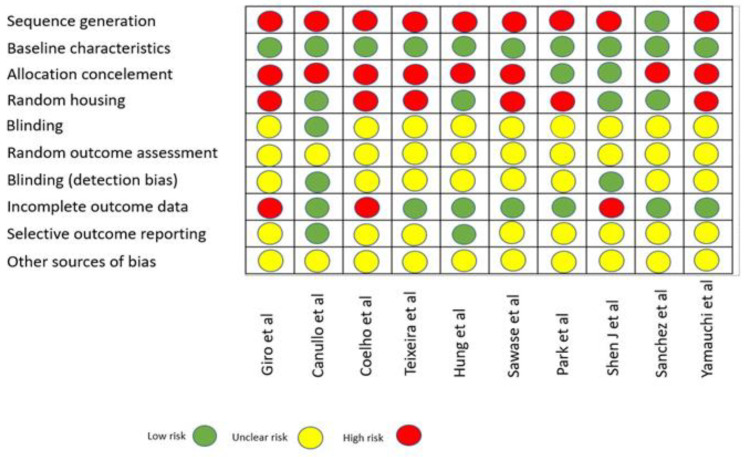
Quality assessment of included animal studies.

According to SYRCLE’s RoB tool (Systematic Review Centre for Laboratory Animal Experimentation), risk of bias of animal studies is assessed and displayed in Figure 2 [19]. No article showed low risk of bias for all domains.

### 3.5. Qualitative Assessment of Outcomes

Differences in bone-to-implant contact (BIC) values between test and control groups were evaluated in eight studies [20,22,23,24,25,26,27,28]. In these studies, the functionalization treatment of the implant surfaces, both by UV and by non-thermal plasma, led to better BIC results than the control group. Only one study in rabbits reported lower BIC values in the test groups (UV-treated) than in the control group [29]. In fact, in the study by Sanchez-Perez et al., at 8 weeks, a minimal variation in the BIC values between the test group and the control group was noted, with BIC values of 26.835% for the control group and 24.225% for the test group. Differences in ISQ values between test group and control group were evaluated in one study [25], in which ISQ values were higher in the test group at 8 and 12 weeks of follow-up compared to the group control. Differences in removal torque values between the test group and control group were evaluated in two studies [20,22]. In all studies the removal torque (Ncm) values were higher in the test groups than in the control groups.

### 3.6. Quantitative Assessment of Outcomes

The meta-analysis was conducted only if at least three articles presented the same treatment method for functionalization of the implant surface, and the same kind of sample and the same outcome of interest (BIC value) was evaluated. Following these criteria, four articles were included in the quantitative evaluation [20,26,27,29], as reported in the PRISMA flow chart (Figure 1). Based on the peculiarities of the follow-up recorded in some of the studies meeting the enrollment criteria (follow-up at different time-points in two studies, with slightly different numbers of implants), the analysis was conducted on multiple datasets identified on the basis of the different follow-up periods in order to even the follow-up time-points, applying the criterion of n = 1 study with unique follow-up for a single dataset. Following this approach, four datasets were assessed. The first dataset (Table S2) took into consideration the following follow-up periods: 2 weeks [26], 4 weeks [27], 3 weeks [20], and 8 weeks [29]. The second dataset (Table S3) resulted in 2 weeks [26], 12 weeks [27], 6 weeks [20], and 8 weeks [29]. The third dataset (Table S4) returned 2 weeks [26], 4 weeks [27], 3 weeks [20], and 8 weeks [29]. Finally, the fourth dataset (Table S5) resulted in 2 weeks [26], 12 weeks [27], 6 weeks [20], and 8 weeks [29].

#### 3.6.1. First Dataset

**Figure 3:**
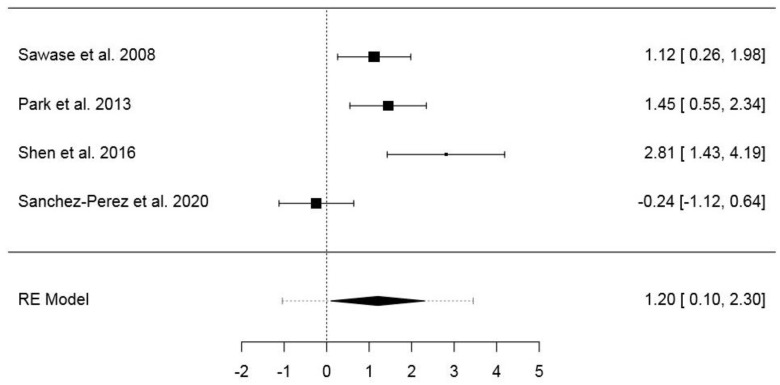
Forest plot for the random-effects model with the DerSimonian–Laird estimator in the first dataset (effects size: standardized mean difference of the bone–implant contact).

The first dataset included 33 animals and 84 implants (Table S2). The pooled SMD of the random-effects model was 1.20 (95% CI: 0.10–2.30) (*p* = 0.032), while the model fit statistics were ML = −5.91 (AIC = 15.82) and RML = −4.87 (AIC = 13.75). The model showed a high heterogeneity (*I*^2^ = 80.33%; *H*^2^ = 5.08; *τ*^2^ = 0.996; *Q* = 15.25; *p* = 0.002). In the normal Q–Q plot, no study was outside the confidence area (Figure S5A). The forest plot is presented in Figure 3. Publication bias assessment returned no statistical significance for Kendall’s Tau (*p* = 0.333) or Egger’s regression (*p* = 0.076) (Figure S9A). The results of the same model with RMD as effect size are presented in the Supplementary Material (Figures S1, S5B and S9B).

#### 3.6.2. Second Dataset

**Figure 4:**
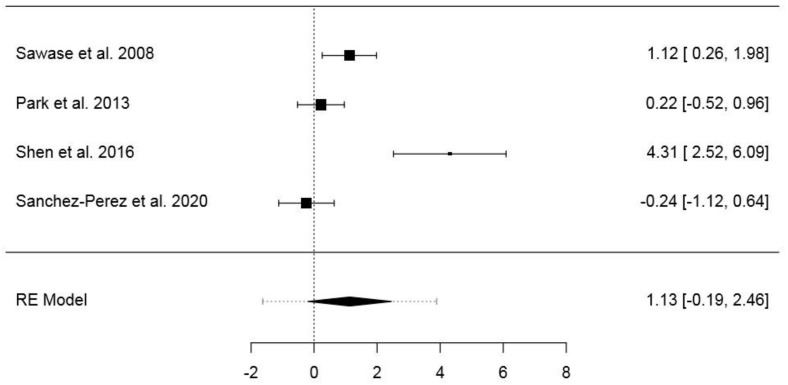
Forest plot for the random-effects model with the DerSimonian–Laird estimator in the second dataset (effects size: standardized mean difference of the bone–implant contact).

The second dataset included 34 animals and 88 implants (Table S3 (Supplementary Materials)). The pooled SMD of the random-effects model was 1.13 (95% CI: −0.19–2.46) (*p* = 0.095), while the model fit statistics were ML = −7.84 (AIC = 19.68) and RML = −6.62 (AIC = 17.24). The model showed a high heterogeneity (*I*^2^ = 86.66%; *H*^2^ = 7.49; *τ*^2^ = 1.524; *Q* = 22.48; *p* ≤ 0.001). In the normal Q–Q plot, one study resulted outside the confidence area (Figure S6A). The forest plot is presented in Figure 4. Publication bias assessment returned no statistical significance for Kendall’s Tau (*p* = 0.750), differently from Egger’s regression (*p* < 0.001) (Figure S10A). The results of the same model with RMD as effect size are presented in the Supplementary Material (Figures S2, S6B and S10B).

#### 3.6.3. Third Dataset

**Figure 5:**
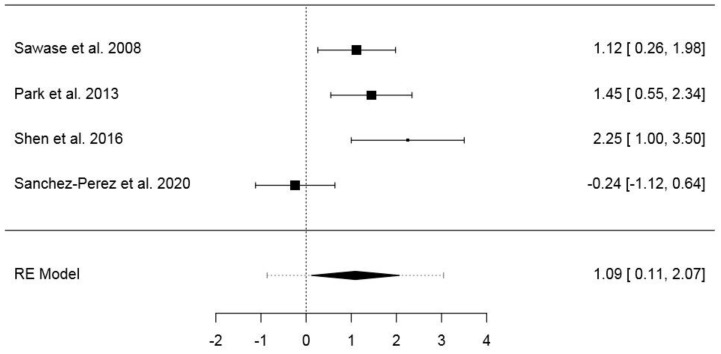
Forest plot for the random-effects model with the DerSimonian–Laird estimator in the third dataset (effects size: standardized mean difference of the bone–implant contact).

The third dataset included 33 animals and 84 implants (Table S4). The pooled SMD of the random-effects model was 1.09 (95% CI: 0.11–2.07) (*p* = 0.028), while the model fit statistics were ML = −5.25 (AIC = 14.51) and RML = −4.34 (AIC = 12.68). The model showed a high heterogeneity (*I*^2^ = 76%; *H*^2^ = 4.17; *τ*^2^ = 0.746; *Q* = 12.50; *p* = 0.006). In the normal Q–Q plot, no study was outside the confidence area (Figure S7A (Supplementary Materials)). The forest plot is presented in Figure 5. Publication bias assessment returned no statistical significance for Kendall’s Tau (*p* = 0.333) or Egger’s regression (*p* = 0.181) (Figure S11A). The results of the same model with RMD as effect size are presented in the Supplementary Material (Figures S3, S7B and S11B).

#### 3.6.4. Fourth Dataset

**Figure 6:**
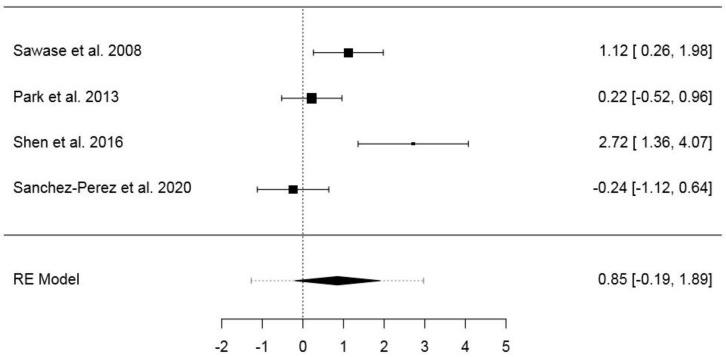
Forest plot for the random-effects model with the DerSimonian–Laird estimator in the fourth dataset (effects size: standardized mean difference of the bone–implant contact).

The fourth dataset included 34 animals and 88 implants (Table S5). The pooled SMD of the random-effects model was 0.85 (95% CI: −0.19−1.89) (*p* = 0.110), while the model fit statistics were ML = −5.98 (AIC = 1.95) and RML = −5.00 (AIC = 13.99). The model showed a high heterogeneity (*I*^2^ = 80.32%; *H*^2^ = 5.08; *τ*^2^ = 0.89; *Q* = 15.24; *p* = 0.002). In the normal Q–Q plot, no study was outside the confidence area (Figure S8A). The forest plot is presented in Figure 6. Publication bias assessment returned no statistical significance for Kendall’s Tau (*p* = 0.750), differently from Egger’s regression (*p* = 0.021) (Figure S12A). The results of the same model with RMD as effect size are presented in the Supplementary Material (Figures S4, S8B and S12B).

## 4. Discussion

This systematic review focused on the evaluation of the effects of the functionalization treatment of the implant surfaces by means of UV or non-thermal Plasma on the peri-implant bone, and, more specifically, it investigated the effects on the biological process of osseointegration, as evaluated by BIC, ISQ (implant stability quotient), and removal torque. The eligibility criteria of the studies were first determined so as to include in the systematic review studies with the following: a minimum number of five animals, which had at least one titanium dental implant; a test group subjected to treatment with UV or non-thermal plasma and a control group; analysis the BIC value with a minimum follow-up of two weeks; and ISQ and/or removal torque. A qualitative analysis of the results was performed on all included studies, and a quantitative analysis was performed on 4 of the 10 included studies.

### Summary of the Results and Possible Limitations

The qualitative analysis of the results shows for the majority of the studies, higher BIC (bone-to-implant contact) values in the test groups subjected to the functionalization treatment of the implant surfaces by UV or by non-thermal plasma. With regard to studies that used UV treatment [20,26,27,28], BIC values were better in the test group when compared to the control group at all the time points. Two studies that used treatment with non-thermal plasma [21,23] did not find significant differences in BIC between the test and the control group at the first follow-up (1 week), but they found significant differences in BIC values between the test and the control group at the second follow-up (3 weeks) with better values for the test group.

The third study evaluating non-thermal plasma treatment [24] showed higher BIC values in the test group than in the control group at both 4 and 8 weeks of healing. Only one study [29] reported slightly higher BIC values for the control group than for the UV-treated test group. Despite this, there is a greater homogeneity of the BIC in the test group compared to the control group.

ISQ values were evaluated in a single animal study [25], demonstrating higher ISQ values in the test group subjected to functionalization treatment with non-thermal argon plasma compared to the control group.

Removal values were evaluated in two studies: one [20] evaluating UV functionalization treatment, and one [22] using functionalization treatment with non-thermal argon plasma. In all these three studies, the removal torque (Ncm) values were higher in the test groups than in the control groups.

The meta-analysis was conducted for four studies [20,26,27,29], in which the test groups were subjected to UV treatment. The assessed outcome was the BIC value. A quantitative analysis of the studies in which the test groups underwent treatment with non-thermal plasma, presenting the same outcome of interest, was not possible due to the lack of data. For the studies included in the quantitative evaluation, following the differences in the follow-up, an analysis was conducted on several datasets identified on the basis of the different follow-up periods without prejudice to the criterion of n = 1 study, and with unique follow-up for each individual dataset [26]. The meta-analysis showed statistically significant difference in favor of the test groups in the first and third dataset, for both SMD and RMD as effect size. No significant differences occurred in the second and fourth dataset. The relatively more homogeneous follow-up of the first and third dataset may have contributed to the model outcomes. However, in all models, a high heterogeneity was found, although the highest *I*^2^ and *Q* values occurred for the second and fourth dataset. Normal Q–Q plots showed overall satisfactory profiles, with all or most of the studies falling within the confidence region in each model. Notably, no significance for publication bias was returned by the models evaluated by entering the first and third dataset. Data from the present meta-analysis confirmed the importance to increase surface energy to stimulate bone formation through the exposition to UV. However, a similar effect was obtained also through the activation of the electronic mantel following plasma of argon bio-activation [49]. Additionally, it must be noted that despite the increase in surface energy, these procedures were proven to not affect the bacterial contamination of the implant surface (or bone augmentation material), confirming to finally result positive for bone growth [58,59,60,61,62].

Limitations can be attributed to the high heterogeneity of the studies, the applied methodology, (including numerosity of the samples, especially in studies on UV treatment), and type of samples tested (dental implants or disks), etc. Different types of devices and time of application were used to functionalize the surfaces of the implants. Three studies [20,26,27] used a source of UV radiation for 24 h; two studies [28,29] used a UV-emitting device for 15 min. However, even if a different application time was employed, all studies obtained better results in the UV-treated groups. The minimum irradiation time required to obtain a clinically appreciable effect is yet to be determined [29]. Three studies [21,22,23] used a plasma device (KinPen Device) for 60 s or for 20 s. The third study [24] used a plasma reactor (Diener electronic) for 12 min. The fourth study [25] used a Plasma reactor (Line through ISO 9001) for 60 s. Further studies should evaluate the better application time of UV and Plasma in order to standardize the technique.

Another limitation is the definition of osseointegration itself, which is a clinical outcome and not a histological one. Surrogates for osseointegration are used to conduct research, for example, BIC and ISQ. These are measures of specific elements of the bone–implant interface; however, there is little evidence that BIC correlates with long-term clinical outcomes of dental implant therapy, and the use of ISQ, although widely used, remains enigmatic [63].

Further limitations are secondary to the incomplete publication of data. BIC and standard deviation values are not reported in three studies [20,21,23] The authors of the aforementioned articles were contacted to supplement the missing data; however, only the BIC values of a single article were obtained [20].

From the clinical point of view, both methods can be used chair-side by the clinician before implant insertion.

Both the investigated techniques of functionalization are easy to apply, cost effective and are devoid of contraindications.

This study and data from animal experiments presented promising outcomes for UV and non-thermal plasma functionalization. An improvement in osseointegration might be expected following biofunctionalization of dental implants. However, it must be emphasized that these results must be taken with caution, as data from animal studies cannot be directly extrapolated to the clinical practice, and the clinical efficacy of these treatments is yet to be established. Human studies are needed to confirm if biofunctionalization of dental implants might affect the bone–implant interface in the short and in the long term.

## 5. Conclusions

Based on the qualitative and quantitative assessment conducted as part of the present systematic review, it can be concluded that the treatment of titanium dental implant surfaces using UV or non-thermal plasma may represent an effective method for improving the osseointegration process. Randomized human studies are needed to validate the obtained results.

## Figures and Tables

**Table 1:** Main characteristics and outcomes of the studies on plasma included.

Study Title	Reference	Year	No of Animals	Specimens	Surface Treatment	Plasma Argon Treatment	Follow-Up	Outcome	Results
Argon-based atmospheric pressure plasma enhances early bone response to rough titanium surfaces	Coelho PG et al. [21]	2012	Six dogs (adult beagle)	Two Ti-6Al-4V implants each side. The different implant surfaces (Ti or Ti-Plasma) were alternately placed from proximal to distal at distances of 1 cm from each other along the central region of the bone.	Alumina- blasted/acid-etched	Atmospheric pressure plasma (CaP-plasma) treatment with Ar gas for a period of 60 s per quadrant with a KinPenTM device	1 and 3 weeks	BIC (%) BAFO (bone area fraction occupacy)	No significant difference was found for BIC and BAFO between surfaces at 1 week. At 3 weeks in vivo, bone formation in close contact to the implant surface (BIC) was strongly observed in the Ti-plasma group, where an increase of over 300% was observed when compared to the control (*p* < 0.001). No significant differences were observed in BAFO (*p* > 0.14), although an improvement of 30% was observed for the Ti-plasma group
Assessment of a chair-side argon-based non-thermal plasma treatment on the surface characteristics and integration of dental implants with textured surfaces	Teixeira H et al. [22]	2012	Six dogs (adult beagle)	Three root-form endosseous grade IV titanium alloy implants placed into each limb. Test: 20 sand 60 s plasma-treated implants; control: untreated implants.	Alumina-blasted and acid-etched surface	Twenty or sixty seconds of non-thermal plasma per quadrant applied with a KinPenTM device	2 and 4 weeks	Removal torque (Ncm)	Torque value at 2 weeks: control: 35; plasma: 20 s—43; plasma: 60 s—55. Torque value at 4 weeks: control: 43; plasma: 20 s—67; plasma 60 s—72.
Osseointegration assessment of chairside argon-based non thermal plasma-treated Ca-P coated dental implant	Giro G et al. [23]	2013	Six dogs (adult beagle)	Two Ti-6Al-4V implants each side. Different implant surfaces were alternately placed from proximal to distal at distances of 1 cm from each other along the central region of the bone, and the start surface site (CaP or CaP-Plasma) was alternated between animals. The implant distribution resulted in an equal number of implants for 1 and 3 weeks.	Calcium-phosphate (CaP)	Ar gas at atmospheric pressure for a period of 20 s per quadrant with a KinPenTM device	1 and 3 weeks	BIC (%) BAFO (bone area graction occupacy)	No significant difference was found for BIC and BAFO between surfaces at 1 week. At 3 weeks, BIC and BAFO were strongly observed in the CaP-plasma group. The morphologic findings for both 1 and 3 weeks were supported by the morphometric results at the 3-week period, as CaP-plasma BIC increased by more than 100% and an improvement of 82% was found for BAFO when compared to the CaP group.
Hard and soft tissue changes around implants activated using plasma of argon: a histomorphometric study in dog	Canullo L et al. [24]	2018	Eight dogs (adult beagle)	For each hemi-mandible, four implants with a ZirTi surface were used; two implants were treated with argon plasma (test), while the other two implants were left untreated (control).	ZirTi surface	Treated for 12 min at room temperature with plasma of argon in a plasma reactor (Diener electronic)	1 and 2 months	BIC (%) old bone total amount of mineralized bone	One month of healing: new bone in close contact with the implant surface: treated (60.1% ± 15.6%; 95% CI 56.5%–78.0%); untreated (57.2% ± 13.1%; 95% CI 49.3%–67.5%) (*p* = 0.400). Old bone: treated (4.4% ± 3.0%; 95% CI. 2%–5.4%); untreated (3.4% ± 3.1%; 95% CI. 6%–4.9%) (*p* = 726). Total amount of mineralized bone: treated (95% CI 59.5%–82.3%); untreated (95% CI 53.3%–73.5%) (*p* = 0.208). Two months of healing of new bone: treated sites: 72.5% ± 12.4% (95% CI 69.6%–86.8%); untreated: 64.7% ± 17.3% (95% CI 59.4%–83.3% (*p* = 0.012). Old bone: treated sites: 3.1% ± 1.7% (95% CI 1.8%–4.2%); untreated sites: 3.8% ± 1.9% (95% CI 3.2%–5.8%) (*p* = 0.270). Total amount of mineralized bone: treated: 75.6% ± 13.0% (95% CI 73.3%–91.3%); untreated 68.4% ± 16.8% (95% CI 64.2%–87.6%).
Effects of non thermal plasma on sandblasted titanium dental implants in beagle dogs	Hung YW et al. [25]	2018	Nine dogs (adult beagle)	Four implants in each dog; control group: one implant withot non-thermal plasma was inserted into each jaw; test group: one implant treated with non-thermal plasma was inserted into each jaw.	Sandblasting and etching	Non-thermal plasma apparatus (Line through ISO 9001) generates plasma in a dielectric barrier. Each implant receive 60s of plasma spray	4,8 and 12 weeks	ISQ Value	ISQ values: Control group: Initial: 68.04 ± 3.37 4 weeks: 66.53 ± 7.40 8 weeks: 69.20 ± 2.55 12 weeks: 74.20 ± 2.68 Plasma group: Initial: 67.36 ± 0.52 4 weeks: 70.17 ± 0.76 8 weeks: 71.50 ± 1.41 12 weeks: 77.00 ± 5.87

**Table 2:** Main characteristics and outcome of included studies about UV.

Study Title	Reference	Year	No of Animals	Specimens	Surface Treatment	UV Treatment	Follow-Up	Outcome	Results
Photo-induced hydrophilicity enhances initial cell behavior and early bone apposition.	Sawase, T et al. [26]	2008	Six rabbits (tibia)	One implant each side of the tibia; cpTi screw implants (Nobel Biocare RP Mark III fixtures; Nobel Biocare AB, Göteborg, Sweden).	Post-annealed from the titanium implant; tetraisoproxide plasma by the plasma source; ion implantation	UV irradiation for 24 h	2 weeks	BIC (%)	BIC untreated: 17.97%; BIC UV: treated 28.2%.
The effect of ultraviolet C irradiation via a bactericidal ultraviolet sterilizer on an anodized titanium implant. A study in rabbits	Park K.H et al. [27]	2013	Fourteen rabbits (tibia)	Twenty-five titanium discs and 56 screw tipe implants. Each rabbits received four control or test implants (UV treated).	Anodized implants	UV irradiation via a 15W lamp for 24 h	4 and 12 weeks	BIC (%)	Four-week mean value: control group (12): 42.92%; test group (12): 55.11%. Twelve-week value: control group (14): 55.81%; test group (14): 57.78%.
The in vivo bone response of ultraviolet-irradiated titanium implants modified with spontaneusly formed nanostructures	Shen J et al. [20]	2016	Forty rabbits (femur and tibia)	A total of 160 screw-shaped implants divided in 5 groups: (1) SLA new (2) SLA old (3) modified SLA (4) UV SLA (5) UV modified SLA.	Sandblasted and acid-etched	UV irradiation via a 15W bactericidial lamp for 24 h	3 and 6 weeks	BIC (%) RT (removal torque)	BIC mean value at 3 weeks: group (1): 40.05% group (2): 30.2% group (3): 35.3% group (4): 59.6% group (5): 61.8% BIC mean value at 6 weeks: group (1): 41.6% group (2): 31.3% group (3): 39.3% group (4): 69.5% group (5): 72.0% Torque removal mean at value 3 weeks: group (1): 42 group (2): 30 group (3): 39 group (4): 70 group (5): 90 Toque removal mean value at 6 weeks: group (1): 70 group (2): 42 group (3): 60 group (4): 82 group (5): 105
Photofunctionalised Ti6Al4V implants enhance early phase osseointegration.	Yamauchi, R et al. [28]	2017	Five rats (femur)	One implant each side; implant made from pure Ti and Ti6Al4V (B. Braun Aesculap Japan Co., Ltd. Tokyo, Japan).	Specimens: pure Ti and Ti6Al4V with average surface roughness values of 0.66 and 0.34 μm, respectively	Exposure to UV irradiation for 15 min using a photo device (TheraBeam Affinity; Ushio Inc., Tokyo, Japan) at an intensity of 3 mW/cm^2^	2 and 4 weeks	BIC value (BV/TV %)	Pure Ti value: Untreated 2-week value: 39.8%; treated 2-week value: 56.8%. Untreated 4-week value: 61.6%; treated 4-week value: 80.7%. Ti6Al4V value: Untreated 2-week value: 44.4%; treated 2-week value: 65.0%; untreated 4-week value: 58.6%; treated 4-week value: 76.3%.
Effects of ultraviolet Photoactivation on osseointegration of commercial pure titanium dental implant after 8 weeks in a rabbit model	Sanchez-Perez A et al. [29]	2020	Five rabbits	Twenty commercial implants.	Group 1: as received; group 2: UV treated	A 6W UVC source for 15 min (Analizer VL 6c)	8 weeks	BIC (%)	BIC mean value: Control group: 26.835%; test group 24.225%.

## Supplementary Materials

The following are available online at https://www.mdpi.com/2077-0383/9/9/2817/s1, Table S1: Research query and their outputs; Table S2: First dataset entered in the meta-analysis; Table S3: Second dataset entered in the meta-analysis; Table S4: Third dataset entered in the meta-analysis; Table S5: Fourth dataset entered in the meta-analysis; Figure S1: Forest plot for the random-effects model with the DerSimonian–Laird estimator in the first dataset (effects size: raw mean difference of the bone–implant contact); Figure S2: Forest plot for the random-effects model with the DerSimonian–Laird estimator in the second dataset (effects size: raw mean difference of the bone–implant contact); Figure S3: Forest plot for the random-effects model with the DerSimonian–Laird estimator in the third dataset (effects size: raw mean difference of the bone–implant contact); Figure S4: Forest plot for the random-effects model with the DerSimonian–Laird estimator in the fourth dataset (effects size: raw mean difference of the bone–implant contact); Figure S5: Normal Q–Q plots related to the random-effects models with the DerSimonian–Laird estimator in the first dataset; Figure S6: Normal Q–Q plots related to the random-effects models with the DerSimonian–Laird estimator in the second dataset; Figure S7: Normal Q–Q plots related to the random-effects models with the DerSimonian–Laird estimator in the third dataset; Figure S8: Normal Q–Q plots related to the random-effects models with the DerSimonian–Laird estimator in the fourth dataset; Figure S9: Funnel plots for the publication bias assessment related to the random-effects models with the DerSimonian–Laird estimator in the first dataset; Figure S10: Funnel plots for the publication bias assessment related to the random-effects models with the DerSimonian–Laird estimator in the second dataset; Figure S11: Funnel plots for the publication bias assessment related to the random-effects models with the DerSimonian–Laird estimator in the third dataset; Figure S12: Funnel plots for the publication bias assessment related to the random-effects models with the DerSimonian–Laird estimator in the fourth dataset.

## Appendix A

**Table A1:** Table reporting the excluded studies and reasons for exclusion.

Article Excluded	Reason for Exclusion
Shon et al., 2013 [30]	Specimens material (zirconia implants)
Naujok et al., 2019 [31]	Sample size
Aita H et al., 2009 [32]	Outcome
Suzuki T et al., 2009 [13]	Outcome
Ueno T et al., 2010 [33]	Outcome
Ueno T et al., 2010 [34]	Outcome
Minamikaw et al., 2014 [35]	Outcome
Hirota M et al., 2016 [36]	Sample size
Ishijima M et al., 2016 [37]	Outcome
Soltanzadeh P et al., 2017 [38]	Outcome
Sugita Y et al., 2014 [39]	Outcome
Taniyama et al., 2020 [40]	Outcome
Jimbo R et al., 2011 [41]	Specimens
Hayashi M et al., 2014 [42]	Specimens
Yamazaki M et al., 2015 [43]	Outcome
Kim H.S et al., 2017 [44]	Farmacological treatment (alendronate)
Lee J.B et al., 2019 [45]	Sample size
Miki T et al., 2019 [46]	Specimens
Hirakawa et al., 2013 [47]	Surface treatment
Pyo et al., 2013 [48]	Sample size
Ishii et al., 2016 [49]	Outcome
Mehl et al. 2018 [50]	Sample size
Funato et al., 2013 [51]	Case series
Suzuki et al., 2013 [52]	Cross-sectional retrospective analysis
Funato et al., 2013 [53]	Retrospective analysis
Kitajima et al., 2016 [54]	Cross-sectional retrospective analysis
Hirota et al., 2016 [55]	Complex cases
Hirota et al., 2018 [56]	Outcome
Puisys et al., 2018 [57]	Specimens
