# Supplementary material for: Photo and Plasma Activation of Dental Implant Titanium Surfaces. A Systematic Review with Meta-Analysis of Pre-Clinical Studies
Source: J Clin Med. 2020 Aug 31;9(9):2817. doi: 10.3390/jcm9092817 (PMC7565759; doi:10.3390/jcm9092817)
Supplement: Supplementary file 1 [file jcm-09-02817-s001.zip › jcm-902341-supplementary.docx]

**ELECTRONIC SUPPLEMENTARY MATERIAL**

**Photo and Plasma activation of dental implant titanium surfaces.**

**A systematic review with meta-analysis**

Paolo Pesce ^1^, Maria Menini ^2^, Gregorio Santori ^3^, Emanuele De Giovanni ^4^, Francesco Bagnasco ^5^ and Luigi Canullo ^6,^*

^1^ Department of Surgical Sciences and Integrated Diagnostics, University of Genoa; paolo.pesce@unige.it

^2^ Department of Surgical Sciences and Integrated Diagnostics, University of Genoa; maria.menini@unige.it

^3^ Department of Surgical Sciences and Integrated Diagnostics, University of Genoa; gregorio.santori@unige.it

^4.^ Department of Surgical Sciences and Integrated Diagnostics, University of Genoa; lele9-90@hotmail.it

^5.^ Department of Surgical Sciences and Integrated Diagnostics, University of Genoa; fcbagna5@hotmail.it

^6.^ Private Practice, Rome; luigicanullo@yahoo.com

| Database | Query | Results |
| --- | --- | --- |
| Pubmed | (((("photofunctionalization"[All Fields] OR "photofunctionalized"[All Fields]) AND "Or"[All Fields] AND ("ultraviolet"[All Fields] OR "ultraviolets"[All Fields])) OR "uv"[All Fields]) OR (((("plasma"[MeSH Terms] OR "plasma"[All Fields]) OR "plasmas"[All Fields]) OR "plasma s"[All Fields]) AND (("argon"[MeSH Terms] OR "argon"[All Fields]) OR "argons"[All Fields]))) AND (("dental implants"[MeSH Terms] OR ("dental"[All Fields] AND "implants"[All Fields])) OR "dental implants"[All Fields] | 220 |
| Scopus | ((photofunctionalization OR ultraviolet OR UV OR (plasma AND of AND argon)) dental AND implants) | 285 |
| Cochrane | ((photofunctionalization OR ultraviolet OR UV OR (plasma AND of AND argon)) dental AND implants) | 13 |

Table S1. Research query and their outputs for each database

| Author | Year | Country | Stype | N.pat | N.imp | N.imp.t | N.imp.c | FU | tmean | tsd | cmean | csd |
| --- | --- | --- | --- | --- | --- | --- | --- | --- | --- | --- | --- | --- |
| Sawase et al. 2008 | 2008 | Japan | histological | 6 | 24 | 12 | 12 | 2 weeks | 28,20 | 10,86 | 17,97 | 6,15 |
| Park et al. 2013 | 2013 | Korea | histological | 6 | 24 | 12 | 12 | 4 weeks | 55,11 | 9,51 | 42,92 | 6,48 |
| Shen et al. 2016 | 2016 | China | histological | 16 | 16 | 8 | 8 | 3 weeks | 59,63 | 11,31 | 30,21 | 8,27 |
| Sanchez-Perez et al. 2020 | 220 | Spain | histological | 5 | 20 | 10 | 10 | 8 weeks | 24,225 | 7,11 | 26,835 | 12,766 |

Table S2: First dataset entered in the meta-analysis

*
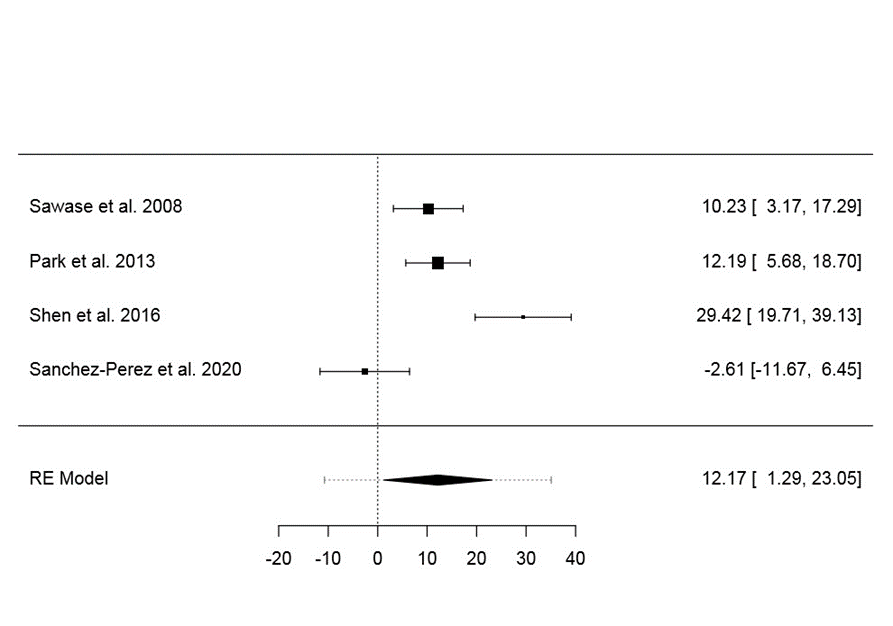
*

**Figure S1**. Forest plot RMD first dataset

| Author | Year | Country | Stype | N.pat | N.imp | N.imp.t | N.imp.c | FU | tmean | tsd | cmean | csd |
| --- | --- | --- | --- | --- | --- | --- | --- | --- | --- | --- | --- | --- |
| Sawase et al. 2008 | 2008 | Japan | histological | 6 | 24 | 12 | 12 | 2 weeks | 28,20 | 10,86 | 17,97 | 6,15 |
| Park et al. 2013 | 2013 | Korea | histological | 6 | 24 | 12 | 12 | 12weeks | 57,78 | 10,49 | 55,81 | 6,53 |
| Shen et al. 2016 | 2016 | China | histological | 16 | 16 | 8 | 8 | 6 weeks | 69,55 | 8,79 | 31,37 | 7,95 |
| Sanchez-Perez et al. 2020 | 220 | Spain | histological | 5 | 20 | 10 | 10 | 8 weeks | 24,225 | 7,11 | 26,835 | 12,766 |

Table S3: Second dataset entered in the meta-analysis

*
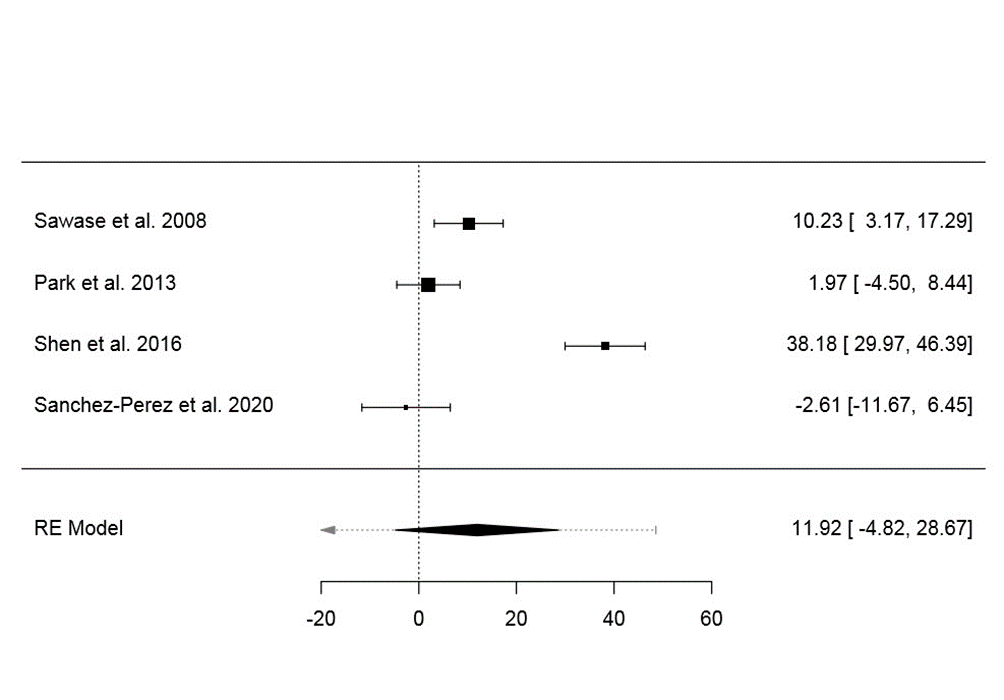
*

**Figure S2.** Forest plot RMD second dataset

| Author | Year | Country | Stype | N.pat | N.imp | N.imp.t | N.imp.c | FU | tmean | tsd | cmean | csd |
| --- | --- | --- | --- | --- | --- | --- | --- | --- | --- | --- | --- | --- |
| Sawase et al. 2008 | 2008 | Japan | histological | 6 | 24 | 12 | 12 | 2 weeks | 28,20 | 10,86 | 17,97 | 6,15 |
| Park et al. 2013 | 2013 | Korea | histological | 6 | 24 | 12 | 12 | 4 weeks | 55,11 | 9,51 | 42,92 | 6,48 |
| Shen et al. 2016 | 2016 | China | histological | 16 | 16 | 8 | 8 | 3 weeks | 61,87 | 11,46 | 35,35 | 10,81 |
| Sanchez-Perez et al. 2020 | 220 | Spain | histological | 5 | 20 | 10 | 10 | 8 weeks | 24,225 | 7,11 | 26,835 | 12,766 |

Table S4: Third dataset entered in the meta-analysis

*
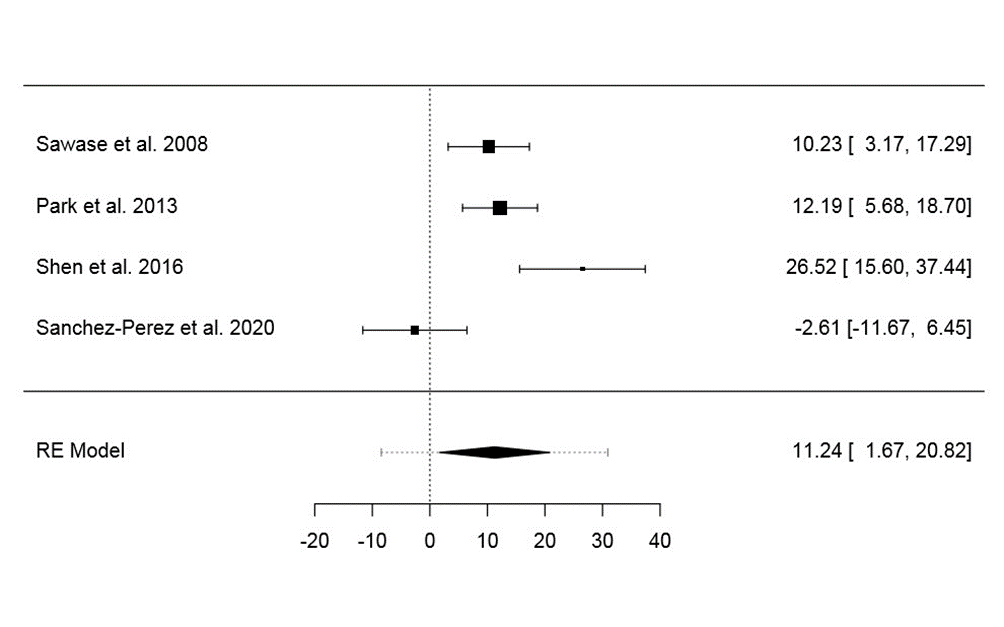
*

**Figure S3.** Forest plot RMD third dataset

| Author | Year | Country | Stype | N.pat | N.imp | N.imp.t | N.imp.c | FU | tmean | tsd | cmean | csd |
| --- | --- | --- | --- | --- | --- | --- | --- | --- | --- | --- | --- | --- |
| Sawase et al. 2008 | 2008 | Japan | histological | 6 | 24 | 12 | 12 | 2 weeks | 28,20 | 10,86 | 17,97 | 6,15 |
| Park et al. 2013 | 2013 | Korea | histological | 6 | 24 | 12 | 12 | 12 weeks | 57,78 | 10,49 | 55,81 | 6,53 |
| Shen et al. 2016 | 2016 | China | histological | 16 | 16 | 8 | 8 | 6 weeks | 72,01 | 11,09 | 39,3 | 11,67 |
| Sanchez-Perez et al. 2020 | 220 | Spain | histological | 5 | 20 | 10 | 10 | 8 weeks | 24,225 | 7,11 | 26,835 | 12,766 |

Table S5: Fourth dataset entered in the meta-analysis

*
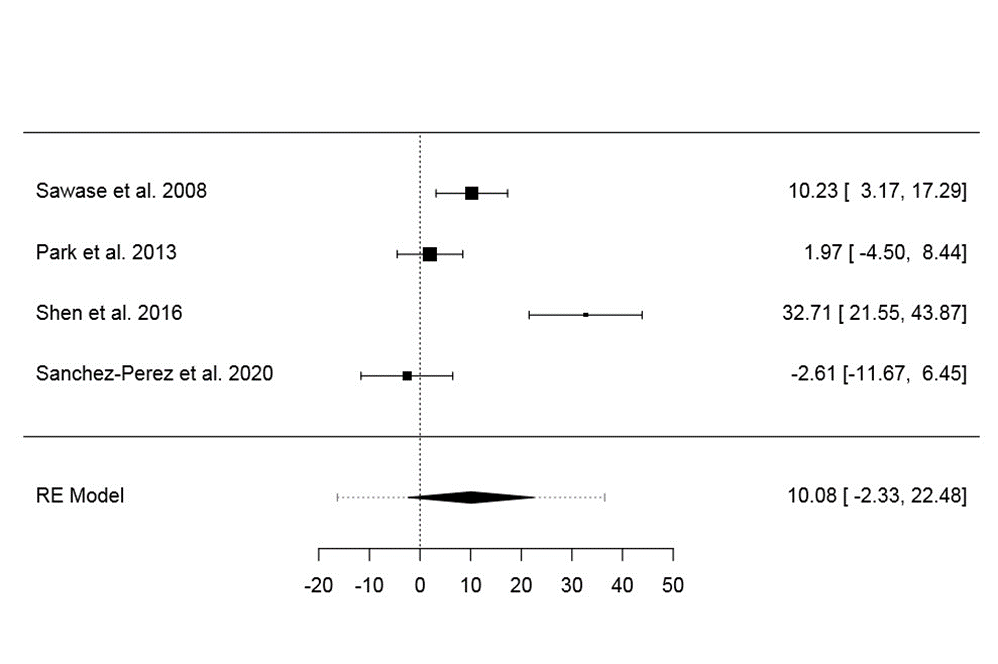
*

**Figure S4.** Forest plot RMD fourth dataset


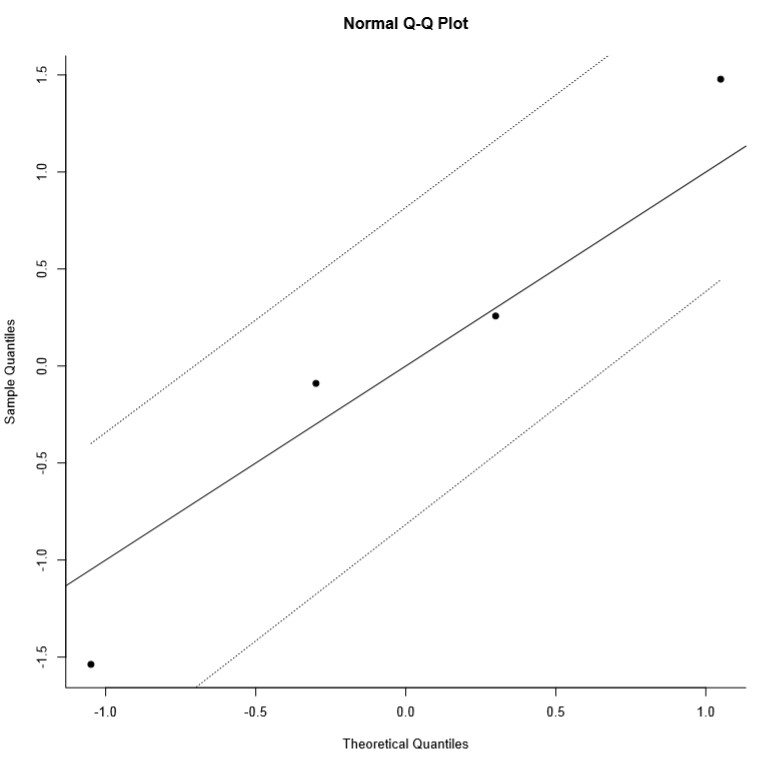


**Figure S5**: Normal Q-Q plots related to the random-effects models with DerSimonian-Laird estimator in the first dataset


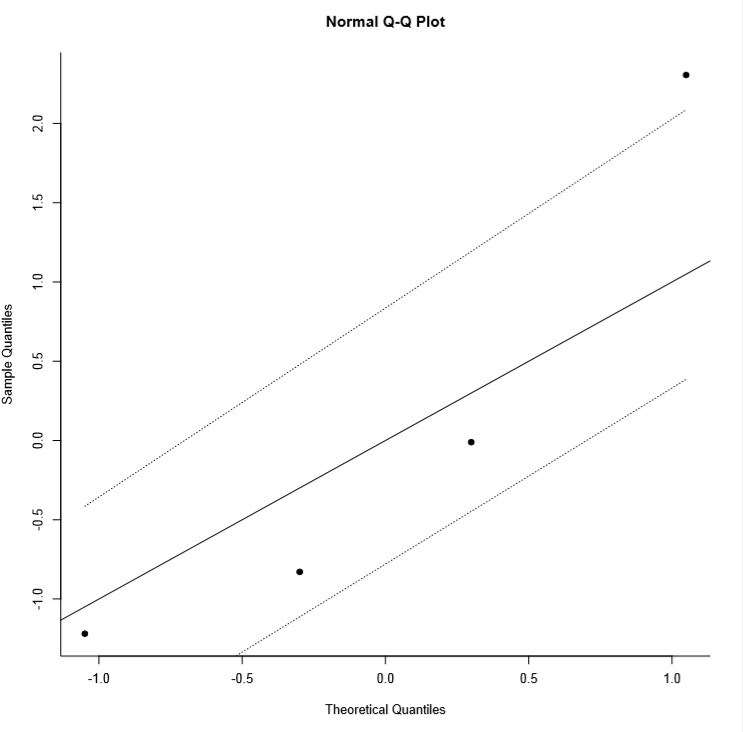


**Figure S6**: Normal Q-Q plots related to the random-effects models with DerSimonian-Laird estimator in the second dataset


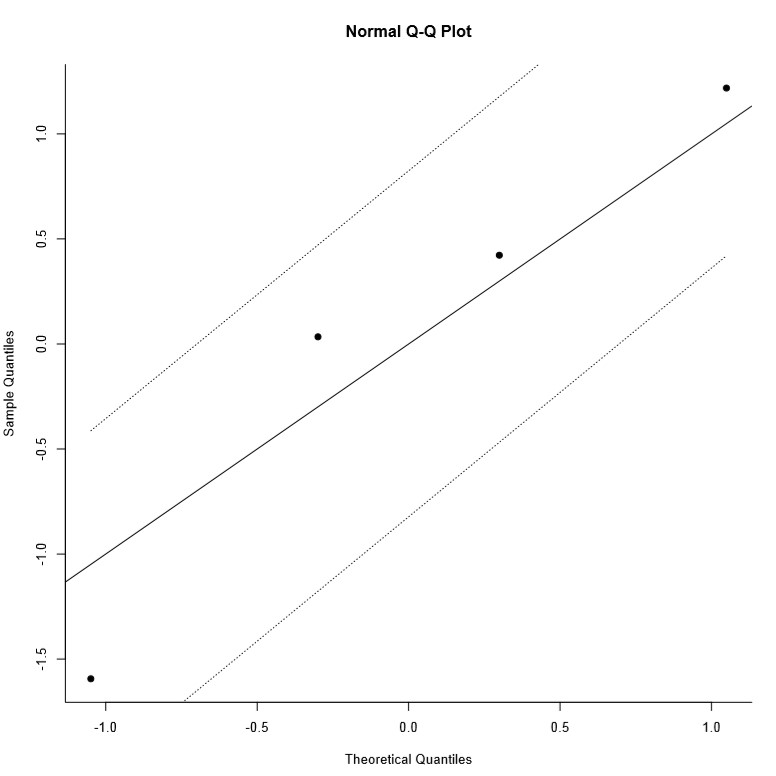


**Figure S7**: Normal Q-Q plots related to the random-effects models with DerSimonian-Laird estimator in the third dataset


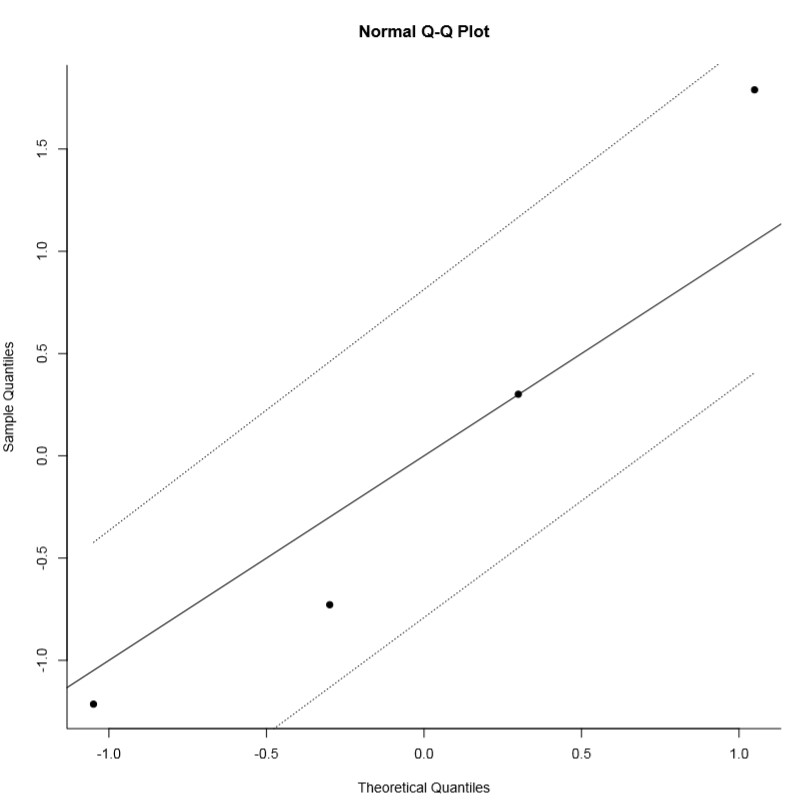


**Figure S8**: Normal Q-Q plots related to the random-effects models with DerSimonian-Laird estimator in the fourth dataset


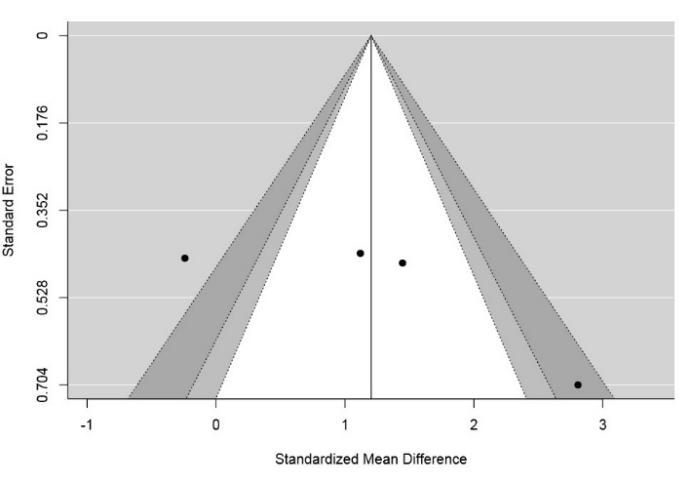

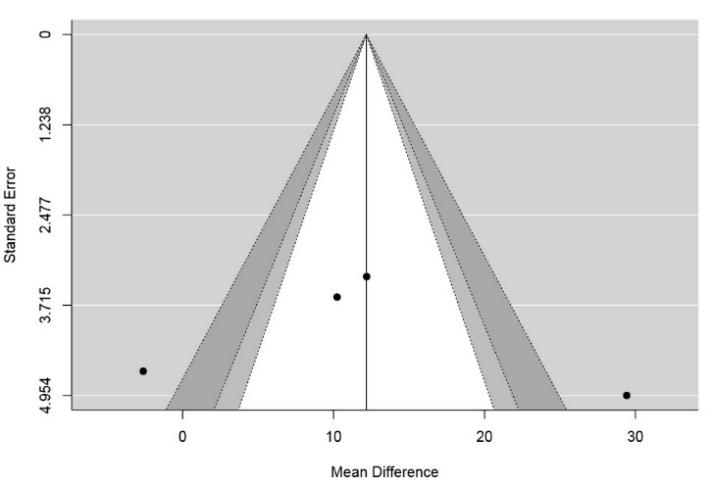


**Figure S9:** Funnel plots for the publication bias assessment related to the random-effects models with DerSimonian-Laird estimator in the first dataset


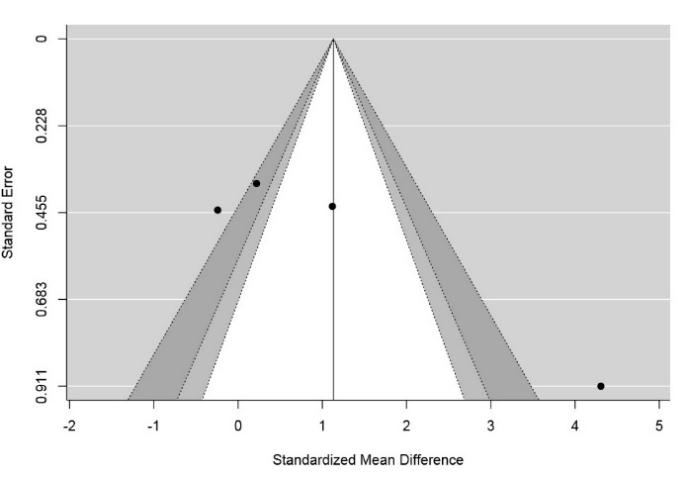

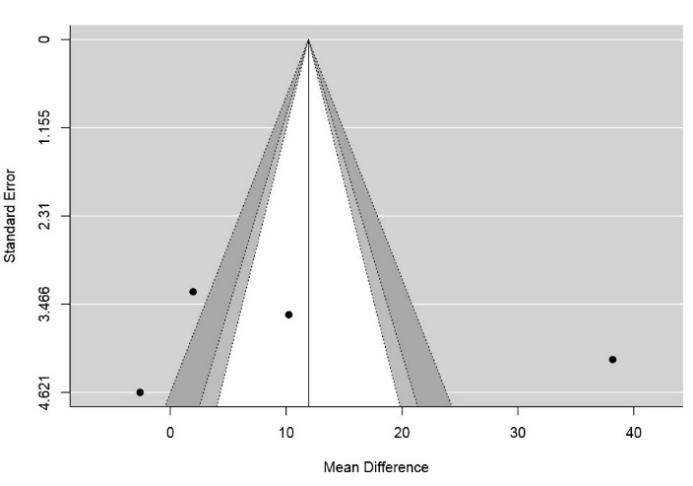


**Figure S10:** Funnel plots for the publication bias assessment related to the random-effects models with DerSimonian-Laird estimator in the second dataset


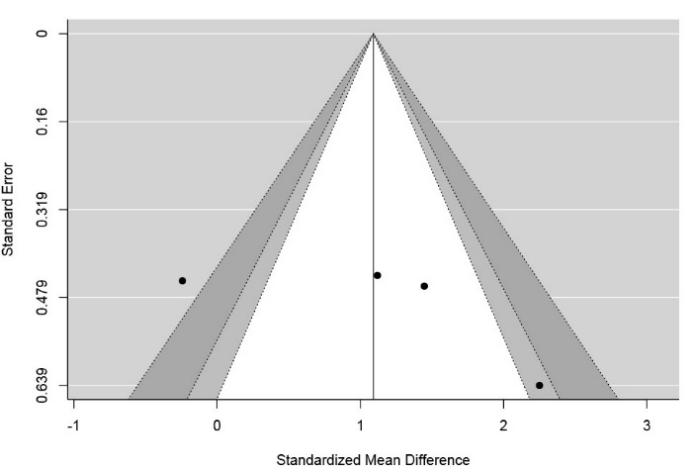

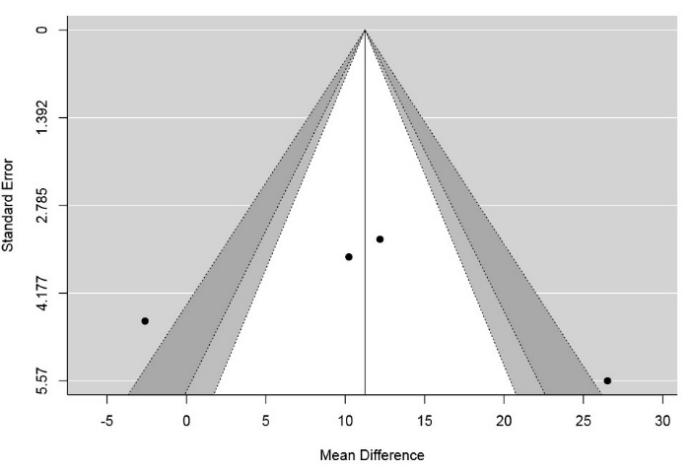


**Figure S11**: Funnel plots for the publication bias assessment related to the random-effects models with DerSimonian-Laird estimator in the third dataset


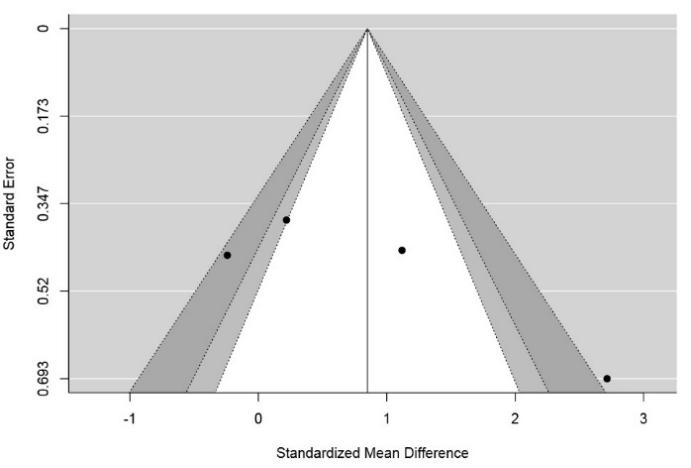

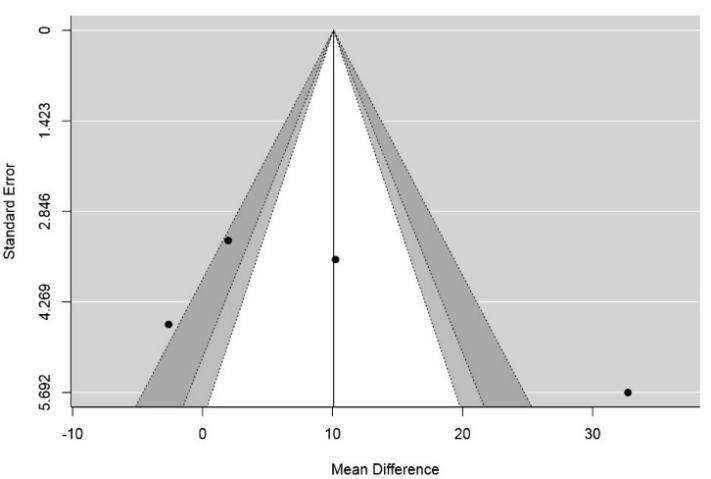


**Figure S12:** Funnel plots for the publication bias assessment related to the random-effects models with DerSimonian-Laird estimator in the fourth dataset
